# Student nurses’ perceptions and experiences in caring for people living with HIV/AIDS: a qualitative study

**DOI:** 10.1186/s12909-023-04074-x

**Published:** 2023-02-07

**Authors:** Chunhong Shi, Jerome V. Cleofas

**Affiliations:** 1 School of Nursing, XiangNan University, Chenzhou, 423000 China; 2 College of Nursing and Allied Health Sciences, St. Paul University Manila, 1004 Manila, Philippines; 3 Department of Sociology and Behavioral Sciences, De La Salle University, 1004 Manila, Philippines

**Keywords:** AIDS, HIV, HIV care, HIV stigma, Qualitative, Perceptions, Nursing students, Nursing education

## Abstract

**Background:**

Caring for people living with HIV/AIDS (PLWHA) requires clinical experience and quality care delivery skills. This study aimed to explore the perceptions and experiences of nursing students in caring for PLWHAs.

**Methods:**

This qualitative descriptive study interviewed 18 student nurses who had cared for PLWHAs from 14 tertiary hospitals across 7 provinces in China through semi-structured telephone interviews.

**Results:**

Two themes emerged from the narratives: student nurses’ perceptions and attitudes toward PLWHAs and student nurses’ practical experiences with PLWHAs. Five theme clusters were revealed, namely “negative attitudes held before the care-giving,” “a series of psychological struggles in care-giving,” “favorable attitudes increased after the care-giving,” “consensus on care delivery for PLWHAs,” and “considerations regarding contamination reduction.”

**Conclusions:**

Findings shed light on the development and changes in student nurses' perspectives on PLWHAs throughout their clinical experiences. Student nurses' perceptions and attitudes toward PLWHAs progressed through three distinct stages, and positive changes were observed after care-giving. Participants' perceptions and practical experiences with patients with AIDS enable patients to receive fair and high-quality care and provide valuable insights for nursing educators better prepare HIV nurses.

**Supplementary Information:**

The online version contains supplementary material available at 10.1186/s12909-023-04074-x.

## Background

Acquired immunodeficiency syndrome (AIDS), caused by the human immunodeficiency virus (HIV), is a chronic and incurable disease spread by sexual intercourse, blood, and mother-to-child transmission [1]. With the rapid increase in the number of people living with HIV and AIDS (PLWHA), HIV and AIDS have become a serious public health and social problem [2]. Healthcare providers may encounter HIV patients in various settings, in addition to the infectious disease sector; it is critical for them to deliver appropriate treatment to this population. However, evidence highlights most healthcare providers have low involvement in HIV prevention and care [3] and report stressors related to providing care for PLWHA [4, 5].

Registered nurses comprise nearly 45% of healthcare providers in China [6], playing an essential role in HIV care. Undergraduate nursing education in China is a four-year program. The clinical internship is a vital transition stage for nursing students to become registered nurses, and it typically lasts 42–52 weeks in China [7]. Internship programs can potentially train and equip better nurses who will serve PLWHAs more effectively. However, previous literature has reported that student nurses lack knowledge about HIV and AIDS, are reluctant to care for PLWHA, are afraid of PLWHA, and exhibit negative attitudes (e.g., homophobia, stigma) toward HIV and AIDS [8, 9]. To address this, education programs and clinical training in HIV/AIDS-related knowledge, precautions, and stigma and discrimination reduction are required.

Previous evidence in China has indicated that educational initiatives that assist nursing students in developing empathy [10], peer interventions combined with standardized patient training [11], and anti-AIDS discrimination training program [12], are essential to delivering patient-centered inclusive care to HIV patients. Internationally, studies have revealed that seminar and virtual patient simulation activity focusing on PLWHAs can positively impact nursing students' attitudes [13, 14]. Although nursing education programs have made great efforts over the past decade, and the proportion of healthcare providers unwilling to take care of patients with AIDS has declined, nursing practitioners still feel fear and tend to keep a distance from PLWHAs [15, 16]. The underlying reasons why HIV and AIDS care occurs in the context of unfavorable nursing attitudes deserve to be futher explored. Student nurses’ feelings and experiences in caring for PLWHAs can help explain the cause and facilitate the development of educational intervention strategies.

The purpose of this paper is to explore the perceptions and experiences of student nurses in caring for PLWHAs in mainland China. To date, we found some cross-sectional quantitative studies investigated nursing students’ knowledge, attitude & belief, and willingness to care toward PLWHAs [17, 18]. Few qualitative studies in mainland China have considered the perceptions and experiences of student nurses in HIV care. As future registered nurses, student nurses represent great potential human resources in caring for and respecting PLWHAs. Preparing every nursing student to become an HIV nurse is essential for addressing the global challenges of HIV care and health services. It is hoped that this paper will inspire effective strategies and practice in nursing educational areas.

## Materials and methods

### Study design

A qualitative study is an ideal design to explore participants' experiences, perceptions, motivations, and behaviors and to answer the whys and hows of the phenomenon [19, 20]. This study adopted a descriptive qualitative methodology to collect data through telephonic interviews, which provided a rich and thick description of the participants' perceptions and attitudes toward PLWHAs before, during, and after care-giving and their practical experience.

### Participant selection

Nursing students who have just finished their clinical internship in general hospitals were recruited through a purposeful sampling method, which allows researchers to choose participants who have knowledge or experiences with the research issue [21]. This sampling strategy was chosen to ensure rich data and a detailed description of the phenomena in qualitative research [22]. The following inclusive criteria were established: (1) full-time undergraduate nursing students; (2) clinical practice duration of more than seven months; (3) have delivered care to one or more PLWHAs; (4) can speak and understand Mandarin Chinese. Students with a history of mental disorders or multimorbidity and part-time students were excluded. We ended recruitment upon reaching data saturation, the point where no new insights emerged from the interviews [23].

### Data collection

After obtaining clearance from the St. Paul University Manila Ethics Committee, a digital recruitment advertisement was distributed on WeChat or QQ. The advertisement explained the inclusion and exclusion criteria, the interview duration, the process, the possible risks and benefits, and the study purpose to potential participants. The eligible participants were invited to contact the researcher by telephone or email. The researcher selected the interviewees purposefully according to variations such as hospital, age, and gender to ensure diversity and obtain rich information about perceptions and experiences. The primary researcher communicated through participant's WeChat and sent the informed consent form to the participant a week prior to the interview, which presented potential interests, risks, study process, and purpose in detail. Participants were asked to read the informed consent form carefully, sign it, and send an e-copy to the researchers before the formal interview.

The original interview outline consisted of two questions and was used to interview two student nurses for the pilot testing. The initial questions were as follows: (1) What do you think about PLWHAs? (2) What were your profound experiences in caring for PLWHAs? After reflecting on the interview process and results, we revised it to form a formal interview outline (Table 1). It consisted of open-ended questions about student nurses' perceptions and experience in caring for PLWHAs. Telephone interviews are easier to establish a harmonious relationship and more convenient and anonymous to interviewees [24]; moreover, they can reduce the interviewer's self-consciousness, prejudice, and stereotypes [25]. Telephone interviews allow more participants to share their experiences and can be a useful alternative to face-to-face interviews [26]. Thus, we utilized telephone interviews to collect data. Telephonic interviews were conducted in a quiet and comfortable environment to prevent being disturbed. During the interview, a voice recorder application was used to record all the interviews and non-verbal information (such as intonation and pauses). The interviewees were encouraged to express their thoughts and perceptions thoroughly. We asked probing questions, such as, "*Can you please elaborate on this?*". Important statements of the interviewees were documented on the paper with the consent of the participants.

**Table 1:** Open questions used to guide in-depth interviews

Contents	Open questions
Attitudes and perceptions	(1) What did you think of persons living with HIV/AIDS (PLWHAs) when you first heard about AIDS? (2) What was your attitude toward PLWHAs when you delivered the care services to them last time? (3) What is your attitude toward PLWHAs now?
Experiences	(1) What were your feelings when you delivered care to PLWHAs? (2) What were your deepest feelings when you were in contact with PLWHAs? (3) What were your concerns when you delivered care to PLWHAs?

### Data analysis

The recorded data were transcribed into transcripts word by word within 24 h or no more than 48 h at the latest after each interview. The interaction process with the interviewees, environmental information, and non-verbal cues were also marked in the transcripts. The general information of the participants was edited into the spreadsheet program. All interviews, transcripts, and data analysis were conducted in Mandarin Chinese. Researchers read and re-read the transcripts carefully, avoided subjective assumptions, and immersed themselves in the interviewees’ statements. We followed a COREQ checklist to report the findings of this study [27].

Colaizzi’s 7 seven-step method for thematic analysis was used to draw insights from the narratives [28], and the qualitative research software Nvivo11 was applied to manage the qualitative analysis process. The specific steps were as follows: (1) Carefully analyzing all the transcripts; (2) Extracting the significant statements and representative content related to the research topic; (3) Refining the recurrent and representative meanings; (4) Summarizing the formulated meanings, found common concepts or characteristics, and formed categories, themes clusters, and themes; (5) Elaborating the topic by adopting the interviewees' perspective; (6) Reporting the essential structure of this discussed phenomenon; (7) Returning the final results to the interviewees for verification. In addition, we sent formulated categories, theme clusters, and themes to participants via WeChat to check if the results matched their perception and experience, and we modified statements based on their feedback (see Table S1 and S2 in supplementary files).

### Trustworthiness

To ensure the rigor of the study, we adhered to the four criteria of dependability, credibility, transferability, and confirmability [29] were adopted as follows: (1) Dependability: The two co-authors who are familiar with Colaizzi's method performed independent analyses of the transcripts. Where their opinions differed, a group discussion was used to compare and examine themes and sub-themes until consensus was reached. (2) Credibility: The interviewers underwent systematic training in the qualitative research theories and methods, techniques of semi-structured interviews, and the report criteria to achieve a better understanding of the qualitative research paradigm. After transcribing, another researcher helped recheck the recording and transcripts to ensure data accuracy, ambiguous sentences, and information. (3) Transferability: Our results were corroborated by two student nurses with experiences of caring for PLWHAs who did not participate in the study but agreed that the findings were consistent with their perceptions and experience in caring for PLWHAs. (4) Confirmability: All research data (e.g., audio recordings, transcripts, text data) were properly stored and organized to facilitate the future checking and validation of results.

## Results

### Demographic characteristics of the participants

From July 16 to August 25, 2021, we purposefully interviewed 18 participants from 14 tertiary hospitals using the maximum variation sampling strategy to achieve in-depth and extensive data [22]. The participants consisted of 4 male students (22.22%) and 14 female students (77.78%), aged 21–23 years (mean age: 22.06 ± 0.73 years). They have delivered care to 1–8 PLWHAs (mean: 2.33 ± 1.61 patients) and received 0–3 times (mean: 1.39 ± 0.92 times) AIDS-related knowledge and skills training. The interview length of the participants was 18–34 min (mean: 24.09 ± 3.69 min). Table 2 shows the demographic information of the participants.

**Table 2:** Socio-demographic characteristics of participants

Participant No	Gender	Age (years)	University	Internship Hospital	Number of PLWHAs Handled	Department	Frequency of participation in AIDS-related training
P1	Female	21	Xiangnan University	Xiangya Second Hospital of Central South University	2	Outpatient department dressing room	2
P2	Female	22	Xiangnan University	Guangdong Zhongshan Bo'ai hospital	2	Surgical department, abortion clinics	1
P3	Female	23	Guangxi University of Chinese Medicine	Nanning Second People's Hospital	2	TCM department, oncology department	2
P4	Female	22	Xiangnan University	Guangdong Zhongshan Bo'ai hospital	8	Gynaecology department	3
P5	Female	22	Guangxi University of Chinese Medicine	People's Hospital of Guangxi Zhuang Autonomous Region	4	Gynecology department, emergency department, neurosurgery department	1
P6	Female	22	Xiangnan University	Chenzhou first people's Hospital	3	Neurology department, thoracic surgery department	1
P7	Female	21	Xiangxing College of Hunan University of Chinese Medicine	The First Affiliated Hospital of Hunan University of Chinese Medicine	2	ICU	1
P8	Female	22	North Sichuan Medical College	Affiliated Hospital of North Sichuan Medical College	1	ICU	1
P9	Female	22	Xiangxing College of Hunan University of Chinese Medicine	Zhuzhou Central Hospital	1	Division of Cardiovascular Medicine	2
P10	Male	22	Xiangnan University	Chenzhou first people's Hospital	2	Cardiothoracic surgery department, neurology department	1
P11	Female	23	Guizhou Medical University	Affiliated Hospital of Guizhou Medical University	2	Delivery room, baby friendly ward	3
P12	Female	22	Kunming Medical University Haiyuan College	The First Affiliated Hospital of Kunming Medical University	1	Emergency Department	0
P13	Female	23	Guangxi University of Chinese Medicine	The First Affiliated Hospital of Guangxi University of Chinese Medicine	2	Department of spleen and stomach, Department of gastrointestinal surgery	2
P14	Male	23	Tibet University	People's Hospital of Tibet Autonomous Region	2	Obstetrics and Gynecology Department	0
P15	Male	21	Hunan University of Chinese Medicine	Zhuhai Hospital of Guangdong Provincial Hospital of Tradtional Chinese Medicine	2	Respiratory department, ICU	2
P16	Female	21	Kunming Medical University Haiyuan College	The Third People's Hospital of Yunnan Province	1	Digestive Department	0
P17	Male	23	Tibet University	People's Hospital of Tibet Autonomous Region	2	Surgical department	1
P18	Female	22	Yunnan University of Bussiness Management	The First Affiliated Hospital of Kunming Medical University	3	Sports medicine department, respiratory medicine department, pediatrics department	2

### Student nurses’ perceptions and experience in caring for PLWHAs

The transcripts of 18 participants were analyzed and categorized into two themes: student nurses' perceptions and attitudes towards PLWHAs and practical experiences in caring for PLWHAs, as indicated in Table 3.

**Table 3:** Themes, theme clusters, and categories emerging from the interviews

Themes	Theme clusters	Categories	No. of responses (*N* = 18)
Student nurses' perceptions and attitudes towards PLWHAs	Negative attitudes held before the care-giving	Feel fear and anxiety about AIDS	14
		Perceived stigma and discriminatory against PLWHAs	9
	A series of psychological struggle in care-giving	Conflict between concerns about occupational exposure and their duty as healthcare workers	16
		Conflict between anxiety and curiosity about caring for PLHWAs	11
	Favorable attitudes increased after the care-giving	Acceptance and empathy	13
		Self-fulfillment	7
Student nurses' practical experiences with PLWHAs	Consensus on care delivery for PLWHAs	Provide fair services to PLWHAs	16
		Improve mental health care of PLWHAs	10
		Protect the privacy and confidentiality of PLWHAs	6
		Establish trusting relationships with PLWHAs	6
	Considerations regarding contamination reduction	Compliance with universal precautions	18
		Assess patient readiness and environmental factors	13
		Receive guidance and strategies from teachers	7
		Improve professional skills in caring services	6

### Student nurses’ perceptions and attitudes towards PLWHAs

#### Negative attitudes held before the care-giving

Before receiving AIDS-related medical knowledge and clinical practice, most student nurses experienced negative attitudes towards PLWHAs, categorized as feel fear and anxiety about AIDS, perceived stigma and discriminatory against PLWHA.

##### Feel fear and anxiety about AIDS

Prior to delivering care for PLWHAs, the majority of participants (*n* = 14) expressed high levels of fear and anxiety about this incurable disease.

Another student stated, "*At that time (in primary school), I thought AIDS was very frightening. I think it is just like a pest. When I hear the word (referring to AIDS), I feel very afraid and then stay away.*" (P18).

Another participant expressed a similar view: "*When I was in junior high school, the teacher showed us those videos [about AIDS], I thought it was terrible. Although it may not be contagious in daily contact, everyone is afraid of… This is an incurable disease. In general, it was very frightening, very scary.*" (P13).

##### Perceived stigma and discrimination against PLWHAs

Half of the participants labeled PLWHA with free sex, homosexual behavior, drug use, and other harmful behaviors, especially before they had undergone specific education about the transmission of AIDS. Discrimination against PLWHA is also common and widespread among student nurses for different reasons.

A student nurse identified PLWHAs as people who could harm society: "*Bad people who take drugs would prick themselves with needles and get infected with HIV*…*Around my life, almost everyone thinks that people living with HIV are bad persons and will bring adverse effects on society. We will keep our distance from HIV-infected people unconsciously.*" (P5).

A participant pointed out, "*Maybe, before I studied medical major, I would avoid PLWHAs, and stay as far away from them as possible*." (P17).

#### A series of psychological struggles in care-giving

Student nurses experienced mixed feelings during the process of caring for PLWHAs. The psychological struggle and conflict in care-giving was another issue discussed by all participants. The following examples demonstrate such conflicts.

##### Conflict between concerns about occupational exposure and their duty as healthcare workers

Because HIV can be transmitted via the exchange of blood, semen, and other bodily fluids of an infected patient, the majority of respondents (8/9) expressed concern about occupational exposure and fear of contracting HIV while participating in the care of PLWHAs. Besides, twelve participants described that it was their duty to care for PLWHAs.

One participant mentioned her fear and worry that she would become infected while caring for PLWHAs, "*I was quite afraid at the beginning because I was afraid that I would accidentally experience needle stick injuries in the process of venipuncture and needle extraction… I still felt a little scared.*" (P16).

A student also mentioned that caring PLWHAs is the responsibility of the nursing staff, "*We should care for all patients, including PLWHAs, with all our heart and soul. We can not ignore them, which is not in compliance with our occupational ethics.*" (P15).

##### Conflict between anxiety and curiosity about caring for PLHWAs

Students voiced that they rarely have the opportunity to contact PLWHAs in college, high school, and junior high school or their residential communities. Eleven participants mentioned that they felt nervous and uneasy in care-giving for PLWHAs. However, three participants clearly stated that they were curious and excited during their first time caring for PLWHAs.

Anxiety was expressed by a student, "*In the process of hospital internship, I feel panic about contacting PLWHAs….very scared, but I have to bear through it and deliver routine care to them.*" (P4).

Another student was curiosity, "*I held a learning attitude at that time. As I entered a new field, I was a little curious about new things. Because I learned this knowledge [how to care for PLWHAs] in books, I seldom met them in my life. I want to know how to care for PLWHAs.*" (P15).

#### Favorable attitudes increased after the care-giving

After caring for PLWHA, participants described a significant transition in their attitudes toward such patients, primarily in terms of acceptance, empathy, and self-fulfillment, and these favorable attitudes significantly contributed to their willingness to care for PLWHA and provide high-quality care.

##### Acceptance and empathy

More than two-thirds of the participants stated that they would not be as afraid of HIV as they once were and would be able to show improved tolerance and acceptance of PLWHA. Half of the participants voiced their compassion and empathy towards PLWHAs and their family members. 

  "*If I meet such patients (PLWHAs) again, I will treat them more peacefully and be more accepting, and I will be less nervous and fearful.*" (P13)

One participant mentioned, "*These people infected with HIV are not accepted on many occasions. Since they suffer from such disease, and their family relationships have been dissolved, they feel abandoned by society; I think they are very pitiful. Eventually, I gradually developed compassion for them."* (P10).

##### Self-fulfillment

Although caring for PLWHAs may make interns feel afraid and anxious, some participants (*n* = 7) described the patient's gratitude, and their positive treatment outcomes increased their self-fulfilling experience.

A student expressed, "*I think I was particularly moved when some PLWHAs would thank us, take our hands, and express their heartfelt gratitude*." (P6).

Similarly, another participant stated, "*I would become courageous to face these patients. During my internship… I think I experienced moderate growth in clinical practice. And I feel less fearful when I am facing them again.*" (P12).

### Student nurses’ practical experiences with PLWHAs

#### Consensus for the improvement of care for PLWHAs

High-quality health care helps reduce the perceived stigma and discrimination among PLWHA and improve their quality of life. Students shared their efficient recommendations on service delivery for PLWHAs, which included five theme clusters: provide fair services to PLWHAs, improve mental health care of PLWHAs, protect the privacy and confidentiality of PLWHAs, establish trusting relationships with PLWHAs.

##### Provide fair services to PLWHAs

People with HIV/AIDS, like other patients, deserve the best care we can provide. Sixteen nursing students repeatedly emphasized the need to understand and accept PLWHA and give fair care to PLWHAs as they do to other patients.

As described by one participant, "*PLWHAs need to be treated like normal patients… Healthcare providers should not bring them (PLWHAs) the impression that they are being treated differently, which could affect their care outcomes.*" (P2).

Participant 18 mentioned, "*Healthcare providers should treat PLWHAs in a normal way, and gradually establish fairness [psychology], treat PLWHAs from the perspective of the non-HIV infected patients and normal persons.*" (P18).

##### Improve mental health care of PLWHAs

PLWHAs have much worse mental health than the overall population, which is often manifested in various psychological disorders such as interpersonal sensitivity, anxiety, depression, hostility, and paranoia. More than half (*n* = 10) of the nursing students expressed healthcare providers need to make more efforts on the psychological problems of PLWHAs and give them mental healthcare.

A student said, "*I feel that people suffering from this disease need psychological instructions from healthcare providers. …This is really important because if no one guides and helps them properly, they will feel that AIDS is very scary…*" (P15).

Similarly, another female participant stated, "*For healthcare providers, giving PLWHAs psychological care is very important; after the patients are infected with HIV, they will be afraid, also endure strange looks and suffer from various psychological problems… I think mental care is also essential for PLWHAs.*" (P18).

##### Protect the privacy and confidentiality of PLWHAs

Privacy and confidentiality are critical issues for PLWHA. One-third of participants mentioned that all relevant stakeholders in healthcare settings should adhere to legal and professional standards of confidentiality or privacy to prevent the disclosure of patient information.

One participant expressed, "*When delivering care to PLWHAs, the first thing is to protect her privacy because PLWHAs are very afraid of being discriminated against by others.*" (P11).

A female participant noted, "*I will protect the patient's privacy more. Because I think the patient's privacy should be protected, especially for PLWHA, because in today's society… Many people hold prejudice against this disease, so I think we should not only protect ourselves but also protect the patient's privacy*." (P12).

##### Establish trusting relationships with PLWHAs

Trust in care-giving is important for PLWHAs and their healthcare providers. In a third of the cases, participants shared experiences building trust and promoting communication with PLWHAs.

A participant stated, "*Usually, we have to deal with PLWHAs well and have a good relationship with them. We need them to cooperate with us. We can talk or chat with them and pay attention to them more often…*" (P4).

Another male participant added, "*I think a trusting relationship – I trust him, I give him therapeutic care operations; He should trust me, tell us his basics conditions.*" (P15).

#### Considerations regarding contamination reduction

How to reduce occupational exposure and contamination in the healthcare environment attracts constant attention from students. Participants summarized important considerations for reducing contamination. This theme included four theme clusters: compliance with universal precautions, receive guidance and strategies from teachers, assess patient readiness and environmental factors, improve professional skills in caring services.

##### Compliance with universal precautions

Healthcare providers always work under the challenge of being contaminated, and they may inadvertently get an infection while taking care of PLWHA. All participants indicated that they followed universal precautions, such as wearing gloves, masks, and protective eyewear when performing procedures that are likely to generate blood or bodily fluids droplets.

Hand hygiene and the use of personal protective equipment (PPE) as a measure to reduce infections were the primary experiences of one participant. "*Follow standard precautions in every operation procedure—wearing gloves, washing hands before and after contacting patients…*" (P11).

Another participant reported: "*I think I should wear gloves, goggles and perform nursing operations on PLWHAs. I'll be more careful… Because of the risk of infection from contact with their bodily fluids …."* (P15).

##### Assess patient readiness and environmental factors

Assessment is a crucial step in the nursing procedure and plays a significant role in nursing practice. Thirteen participants indicated the need to assess themselves, the patient, and the environment to reduce the possibility of occupational exposure when providing services to PLWHAs, such as whether they had any wounds, how cooperative the patient was, and whether the environment was bright and spacious.

A participant stated, "*Before contacting each patient, we should check whether there is any special situation and whether he has infectious diseases, and then take care of them and protect ourselves*" (P11).

Another female respondent added, "I'm *afraid of occupation exposure. When I pull needles for PLWHAs, I am very careful and pay attention to whether people are moving around. Assess the patient's current mood and attention. For example, if he is on the phone and not fully involved in my operation, occupational exposure could easily occur, which is very dangerous.*" (P3).

##### Receive guidance and strategies from teachers

Student nurses are aware of their lack of working experience in caring for PLWHA and need to seek guidance and strategies from clinical teachers to cope with the challenges of managing for PLWHA and to protect themselves from the risk of infection. Seven participants clearly expressed a need for guidance from the clinical instructor.

One participant pointed out: "*When we participate in the practice of PLWHA, our teachers will remind us of the patients' condition before providing care. The nurses will also mark PLWHAs red in the medical records and shift books*." (P5).

The need for guidance on occupational exposure prevention was also expressed. "*When I felt afraid, I seek advice from the teacher. When conversing with them, I mentioned the concern about occupational exposure related to AIDS care…The teachers have a lot of experience. After talking with them, I was able to draw insights regarding what they were doing and what they thought of AIDS*." (P16).

##### Improve professional skills in caring services

Care delivery is a complex procedure requiring both knowledge and skills. Occupational exposure is more likely to occur in nurses who have a low level of knowledge about needlestick injury prevention and do not receive relevant training or education. Six students indicated that improving their professional skills was one way to reduce the likelihood of occupational exposure.

A student recounted the importance of improving puncture technique: *"As a nurse, the first thing is to have good needle technique… I need to constantly improve their puncture techniques and perform some routine nursing operations so that we can provide care to PLWHAs smoothly and fearlessly*." (P10).

Another student noted, "*…The most important thing we should do is improve our skills… with good puncture technique."* (P15).

## Discussion

We observed the positive changes among student nurses who had provided nursing for PLWHA, from negative attitudes and misconceptions to struggling and conflicting psychology in care-giving, and then to positive perceptions after care-giving.

Before care-giving, student nurses presented fear, stigma, and discrimination toward HIV-infected patients, which may impact patient care [30, 31]. This is consistent with previous research that people are often afraid of contracting what they perceive as a deadly virus [32] and may choose to avoid exposure to PLWHAs because of a lack of information about HIV transmission [33]. HIV stigma is prevalent in Chinese collectivistic culture [34] and has been identified as a major barrier to HIV prevention and treatment [35]. Consistent with our findings, Reinius et al., [36] and Mahamboro et al., [37] suggest that healthcare providers often exhibit discrimination and prejudice against PLWHAs within communities and healthcare settings. Stigma and discrimination from healthcare practitioners raise the likelihood of unwillingness to seek help and infection spreading in the community [38, 39], threatening the attainment of WHO's ambitious vision 2030 of 90:90:90 for HIV/AID.

During care-giving, student nurses demonstrated a conflict between concerns about occupational exposure and their duty as healthcare workers, as well as a conflict between anxiety and curiosity about caring for PLHWAs. Healthcare providers are generally concerned about occupational exposure while caring for PLHWAs, particularly fear of needlestick injuries, which also aligns with previous findings [40, 41]. Mashallahi and her colleagues [15] also concluded that nurses in Ulmia are constantly afraid of being infected with viruses while caring for patients, which may make healthcare providers hesitant to engage in providing care to PLWHAs and refuse to provide care [42]. Furthermore, "sense of duty" emerged as another category from the transcripts, consistent with Karuppiah's [43] findings. Nurses are responsible for providing unbiased nursing services while adhering to the requirements of the nurses' code of ethics. When student nurses provided care to HIV patients for the first time, they felt nervous and anxious, as they were unconfident in their abilities and unconvinced that they were taking appropriate measures to prevent potential infection [44]. Inspiringly, students demonstrate curiosity and thirst for new knowledge, which is considered a prerequisite for knowledge construction and is associated with active learning [45].

Favorable attitudes increased after care-giving. Categories of "tolerance and acceptance," "empathy," and "self-fulfillment" were shared by student nurses. The experience of caring for PLWHA led to more tolerant attitudes and greater acceptance related to the disease [36]. Proximity to and frequent contact with PLWHA appears to nurture empathy, similar to the results of Mrcem, Ghani, & Post [46]. Nursing students' empathy seems to be linked to their willingness to care for PLWHAs [10]. Participants shared their feelings of "self-fulfillment," and they were proud of their work when the patient recovered from their sufferings, consistent with previous findings [47]. Nurses are often proud of nursing behaviors and their work as wingless angels in the fight against diseases.

These findings demonstrate a practical approach for nursing educators to foster positive beliefs and perceptions among student nurses by alleviating their concerns about professional exposure and increasing their professional identity. Furthermore, more HIV-focused training interventions in clinical settings, such as experiential learning, work with PLWHA, and narrative photography, are recommended to facilitate students' positive attitudes toward PLWHAs.

Student nurses also shared positive and effective practical experiences in HIV & AIDS services. These experiences not only enable patients to receive fair and high-quality care but also better protect the occupational exposure of healthcare providers.

Most participants mentioned the "provide fair services to PLWHA" category. PLWHAs have the same rights as other citizens, regardless of ethnicity, gender, sexual orientation, religion, or financial situation, with the right to equal person-centered care and treatment [48, 49]. Furthermore, student nurses observed patients' psychological needs and the importance of maintaining HIV patients' mental health, which healthcare providers in clinical settings frequently overlook. PLWHAs have a high prevalence of mental disorders and emotional comorbidities, as high as 78.8% [50], and there is an urgent need for comprehensive treatment and mental health services. Student nurses raised the ethical consideration of protecting the privacy and confidentiality of PLWHAs, which is consistent with medical ethics [48, 51]. Violations of confidentiality frequently result in patients discontinuing treatment. "Establishing trusting relationships with PLWHAs" was also an important experience for participants, in coincide with previous research [52, 53]. A trusting and respectful nurse-patient relationship frequently has a positive impact on patient health outcomes.

Another issue for student nurses is how to reduce potential occupational exposure. Contamination reduction considerations were the most important experiences and feelings of student nurses in caring for PLWHAs, which is congruent with the findings of previous studies [54]. Compliance with universal precautions, such as wearing personal protective equipment (PPE), is a critical issue in care-giving, which protects nurses and allows them to provide quality care [55]. Our participants also discussed the necessity of assessing patient readiness and environmental factors. Accurate assessment of environmental and personal background and physical and mental health can assist nurses in gathering objective data and being well-prepared to meet patients' clinical needs and practice management. Besides, obtaining guidance and strategies from teachers is another category in this study. Clinical mentors' instructions allow student nurses to follow their practices and minimize students' occupational contamination [56, 57]. Improving professional skills is also mentioned to address the challenges of caring for PLWHA. Transitioning to registered nurses, student nurses frequently experience a period of lacking clinical skills [58] and are unsure of their ability to meet the needs of HIV patients. Extensive strategies, such as adherence to universal precautions, assessment of patient preparedness and environmental factors, seeking teacher guidance and strategies, as well as improving professional skills, can assist students in minimizing occupational exposure.

### Limitations

This study conducted telephone interviews with a small sample (18 undergraduate student nurses); hence, the transferability of the research results might be limited. However, we recruited participants from a wide range of hospitals (14 tertiary hospitals) to enrich the results. Two student nurses who did not participate in the study confirmed our findings to broaden transferability. Telephone interviews limited the capture of body language. Face-to-face interviews or online interviews may make up for this defect. Qualitative research has inherent limitations, such as the subjective tendency in the process of data analysis. Nonetheless, the results of this study were verified by the participants. The interview outline was not validated by experts and may have potential bias, although the pilot testing among two students was conducted. The interviews explored the students' perception of and experience in AIDS care without consideration of the experience of other stakeholders. Further research can explore the perspectives of registration nurses, patients, and nursing educators, which may provide further insight into HIV-related care and training.

## Conclusions

Our study sheds light on student nurses’ changing attitudes and perceptions before, during, and after caring for PLWHAs, as well as essential recommendations for practice improvement relating to HIV care and contamination reduction. From the narratives of the student nurses, five theme clusters emerged, namely “negative attitudes held before the care-giving,” “a series of psychological struggles in care-giving,” “favorable attitudes increased after the care-giving,” “consensus on care delivery for PLWHAs,” and “considerations regarding contamination reduction.” Our findings could help nursing educators prepare young nurses to care for PLWHAs. To facilitate students’ positive attitudes toward PLWHAs, nursing educators and clinical instructors could offer more opportunities for students to work with PLWHAs in various clinical settings. Recommendations such as an internship program in the AIDS wards and contamination-reduction training programs are identified in the present findings and can inform initiatives to address the challenges of student nurses responding to the multiple needs of HIV patients. Future research may consider examining the extent of the application of the recommended approaches in nursing education and healthcare practice.

## Supplementary Information

**Additional file 1:** Sample tables demonstrating the qualitative analytic processes.

## Data Availability

The datasets used and/or analyzed during the current study available from the corresponding author on reasonable request.

## Abbreviations

HIV: Human immunodeficiency virus
AIDS: Acquired immunodeficiency syndrome
PLWHA: People living with HIV & AIDS
UNAIDS: The Joint United Nations Programme on HIV & AIDS
WHO: World Health Organization
China CDC: Chinese Center for Disease Control and Prevention
