# Supplementary material for: Student nurses’ perceptions and experiences in caring for people living with HIV/AIDS: a qualitative study
Source: BMC Med Educ. 2023 Feb 7;23:99. doi: 10.1186/s12909-023-04074-x (PMC9906868; doi:10.1186/s12909-023-04074-x)
Supplement: Supplementary file 1 — Additional file 1: Sample tables demonstrating the qualitative analytic processes. [file 12909_2023_4074_MOESM1_ESM.docx]

**Table S1: Examples of the Process of Creating Formulated Meanings from Significant Statements.**

| **Significant Statements** | **Formulated Meanings** |
| --- | --- |
| “*At that time (in primary school), I thought AIDS was very frightening. I think it is just like pest. When I hear the word (referring to AIDS), I feel very afraid and then stay away*” (Transcript 18, page 1, lines 6-8). | AIDS is frightening, and students feel very afraid of it |
| “*Bad people who take drugs would prick themselves with needles and get infected with HIV...Around my life, almost everyone thinks that people living with HIV are bad persons and will bring adverse effects on society. We will keep our distance from HIV infected people unconsciously*.” (Transcript 5, page 1, lines 9-16). | PLWHA are bad people with bad behaviors |
| “*Maybe, before I studied medical major, I would avoid PLWHAs, and stay as far away from them as possible.*” (Transcript 17, page 1, lines 16-17). | Students tend to keep away from PLWHAs |
| “*I was quite afraid at the beginning, because I was afraid that I would accidentally experience needle stick injuries in the process of venipecture and needle extraction... I still felt a little scared.*” (Transcript 16, page 2, lines 39-40). | Students concern about needle stick injuries |
| “*Because we are engaged in the nursing occupation. We should care for all patients, including PLWHAs, with all our heart and soul. We can not ignore them, which is not in compliance with our occupational ethics*.” (Transcript 15, page 10, lines 207-211). | Caring for PLWHAs is an occupational duty |
| “*In the process of hospital internship, I feel very panic about contacting PLWHAs....very scared, but I have to bear through it and deliver routine care to them.*” (Transcript 4, page 1, lines 7-9). | Students feel nervous about caring for PLWHAs |
| “*At that time, I held a learning attitude. As I entered a new field, I was a little curious about new things. Because I learned this knowledge [how to caring for PLWHAs] in books, I seldom met them in my life. I want to know how to care for PLWHAs.*” (Transcript 15, page 2, lines 36-38). | Students are curious about caring for PLWHAs |
| “*If I meet such patients (PLWHAs) again, I will treat them more peaceful and accepting, and I will be less nervous and fearful.*” (Transcript 13, page 1, lines 13-14). | Students will accept PLWHAs in future work |
| “*These people infected HIV are not accepted on many occasions. Since they suffer from such disease, their families dissolved, and then they feel being abandoned by the society; I think they are very pitiful, I slowly develop compassion for them.*” (Transcript 10, page 7, lines 146-148). | Students feel empathy towards PLWHAs after care-giving |
| “*I think I was particularly moved when some PLWHAs would thank us, take our hands, and express their heartfelt gratitude.*” (Transcript 6, page 5, lines 106-107). | Students feel moved after care-giving |

**Table S2: Example of How the First Theme “Student nurses’ perceptions and attitudes towards PLWHAs” Was Constructed From Different Clusters of Themes and Formulated Meanings.**

| **Examples of Formulated Meanings** | **Categories** | **Theme Clusters** | **Theme** |
| --- | --- | --- | --- |
| AIDS is frightening, and students feel very afraid of it | Feel fear and anxiety about AIDS | Negative attitudes held before the care-giving | Student nurses’ perceptions and attitudes towards PLWHAs |
| PLWHA are bad people with bad behaviors | Perceived stigma and discriminatory against PLWHA |  |  |
| Students tend to keep away from PLWHAs |  |  |  |
| Students concern about needle stick injuries | Conflict between concerns about occupational exposure and their duty as healthcare workers | A series of psychological struggle in care-giving |  |
| Caring for PLWHAs is an occupational duty |  |  |  |
| Students feel nervous about caring for PLWHAs | Conflict between anxiety and curiosity about caring for PLHWAs |  |  |
| Students are curious about caring for PLWHAs |  |  |  |
| Students will accept PLWHAs in future work | Acceptance and empathy | Favorable attitudes increased after the care-giving |  |
| Students feel empathy towards PLWHAs after care-giving |  |  |  |
| Students feel moved after care-giving | Self-fulfillment |  |  |
